# Optimizing *b*‐values schemes for diffusion MRI of the brain with segmented Intravoxel Incoherent Motion (IVIM) model

**DOI:** 10.1002/acm2.13986

**Published:** 2023-04-09

**Authors:** Chiara Paganelli, Marco Andrea Zampini, Letizia Morelli, Giulia Buizza, Giulia Fontana, Luca Anemoni, Sara Imparato, Giulia Riva, Alberto Iannalfi, Ester Orlandi, Guido Baroni

**Affiliations:** ^1^ Department of Electronics, Information and Bioengineering Politecnico di Milano Milan Italy; ^2^ MR Solutions Americas LLC Acton (MA) USA; ^3^ Bioengineering Unit, National Center of Oncological Hadrontherapy (CNAO) Pavia Italy; ^4^ Radiation Therapy Technologist Unit National Center of Oncological Hadrontherapy (CNAO) Pavia Italy; ^5^ Radiology Unit National Center of Oncological Hadrontherapy (CNAO) Pavia Italy; ^6^ Clinical Department National Center of Oncological Hadrontherapy (CNAO) Pavia Italy

**Keywords:** brain, *b*‐values optimization, DWI, IVIM

## Abstract

**Purpose:**

To define an optimal set of *b*‐values for accurate derivation of diffusion MRI parameters in the brain with segmented Intravoxel Incoherent Motion (IVIM) model.

**Methods:**

Simulations of diffusion signals were performed to define an optimal set of *b*‐values targeting different perfusion regimes, by relying on an optimization procedure which minimizes the total relative error on estimated IVIM parameters computed with a segmented fitting procedure. Then, the optimal *b*‐values set was acquired in vivo on healthy subjects and skull base chordoma patients to compare the optimized protocol with a clinical one.

**Results:**

The total relative error on simulations decreased of about 40% when adopting the optimal set of 13 *b*‐values (0 10 20 40 50 60 200 300 400 1200 1300 1400 1500 s/mm^2^), showing significant differences and increased precision on D and f estimates with respect to simulations with a non‐optimized *b*‐values set. Similarly, in vivo acquisitions demonstrated a dependency of IVIM parameters on the *b*‐values array, with differences between the optimal set of *b*‐values and a clinical non‐optimized acquisition. IVIM parameters were compatible to literature values, with *D* (0.679/0.701 [0.022/0.008] ·10^−3^mm^2^ */*s), *f* (5.49/5.80 [0.70/1.14] %), and D* (8.25/7.67 [0.92/0.83] ·10^−3^mm^2^ */*s) median [interquartile range] estimates for white matter/gray matter in volunteers and *D* (0.709/0.715/1.06 [0.035/0.023/0.271] ·10^−3^mm^2^ */*s), *f* (7.08/7.84/21.54 [1.20/1.06/6.05] %), and D* (10.85/11.84/2.32 [1.38/2.32/4.94] ·10^−3^mm^2^ */*s) for white matter/gray matter/Gross Tumor Volume in patients with skull‐base chordoma tumor.

**Conclusions:**

The definition of an optimal *b*‐values set can improve the estimation of quantitative IVIM parameters. This allows setting up an optimized approach that can be adopted for IVIM studies in the brain.

## 1 INTRODUCTION

The concept of Intravoxel Incoherent Motion (IVIM) was introduced by Le Bihan^1^, ^2^ to separate and quantify the microcirculatory effects from thermal diffusion in Diffusion Weighted Magnetic Resonance Imaging (DWI).

IVIM has gained great interest in the clinical routine as it does not require an exogenous contrast agent to be injected and provides perfusion and diffusion information simultaneously, by fitting the signal to a biexponential model.^2^ Indeed, IVIM shows great potential in many applications in neuro‐oncology and the study of neurological and neurovascular diseases, including the diagnosis and decision‐making in acute strokes,^3^ the differentiation,^4^ prognosis,^5^ and progression of gliomas, as well as differentiation of meningiomas.^6^, ^7^ IVIM parameters have been also recently investigated as biomarkers in targeted therapies for treatment response assessment and early prediction of progression. In this context, studies have been conducted on glioblastoma treated with chemoradiation^8^ and on tumor progression in brain metastases treated with stereotactic radiotherapy.^9^ Recently, the use of daily IVIM in newly commercial MRI‐linac units^10^ and in advanced charged particle therapy^7^, ^11^, ^12^ have been investigated, confirming its growing potential in the clinical and research communities.

Despite the interest, no consensus on a standard IVIM acquisition protocol has been established. This results in dispersed reference values of IVIM parameters’ estimates, which are possibly mingled with uncertainties and inaccuracies,^13^ thus hindering the comparison of results reported in the literature. The optimization of acquisition parameters has the potential to reduce errors in parameters’ estimation^14^, ^15^ and thereby either to reduce scan time or to improve the quality of IVIM parametric maps. Specifically, the computation of diffusion and perfusion parameters in IVIM relies on the possibility to encode the effects of molecular diffusion by playing gradients with different lengths (or amplitudes) during image acquisitions. Diffusion gradients characteristics are summarized in the *b*‐value (s/mm^2^), which depends on the diffusion gradient waveform, the time duration of the gradients and the interval between them. Different studies in the literature investigated the effect of selecting different *b*‐values on DWI acquisitions,^15^, ^16^ with some of them implementing optimization frameworks to automatically derive the optimal set. Specifically, optimal choices of *b*‐values have been widely studied for the mono‐exponential diffusion model,^17^, ^18^ and some studies have implemented similar optimization strategies on the IVIM model for applications in kidney, liver, and breast.^19^, ^20^, ^21^, ^22^ In brain, a recent study by Chabert and colleagues^14^ has been conducted on volunteers, showing that an optimized *b*‐value sampling scheme improves the estimation of IVIM parameters in a low‐perfused organ in comparison with a regular literature based *b*‐value sampling scheme.

In this study we investigated an optimal set of *b*‐values for IVIM acquisitions in the brain, by exploiting an optimization framework based on the minimization of the total relative error of the estimated IVIM parameters computed with a segmented fitting procedure, similarly to Lemke et al.^23^ To mimic different brain compartments as patho‐physiological conditions, we investigated optimal set of *b*‐values according to different perfusion regimes (i.e. low, medium, and high). The work included in‐silico Monte‐Carlo simulations for the definition of the optimal set of *b*‐values, and in vivo acquisitions on healthy volunteers and patients with skull base chordoma tumor acquired at the National Center for Oncological Hadrontherapy (CNAO, Italy, Pavia).

## 2 MATERIAL AND METHODS

### 2.1 Simulated data

#### 2.1.1 Data generation

Simulations were performed to develop and evaluate a general framework for the optimization of the set of *b*‐values employed to estimate IVIM parameters. All simulations and computations were performed in MATLAB R2019b (Mathworks Inc., Natick, MA). The IVIM model was described by the following equation^1^, ^2^:

(1) $$\frac{S\left(b\right)}{S\left(0\right)}=fe^{-bD^{*}}+\left(1-f\right)e^{-bD}$$

where *f* is the flowing blood fraction (perfusion fraction), *D* is the water diffusion coefficient in the tissue, D* is the pseudodiffusion coefficient that includes blood flow and water diffusion in the blood. Three literature‐based parameters’ sets of *D*, *f* , and D* were used to simulate the biexponential signal intensity decay curves of the IVIM model (Equation 1). The three parameters’ sets are mainly characterized by different values related to the perfusion regime. Specifically, we defined^20^, ^24^, ^25^:

- Low perfusion as *D* = 1·10^−3^ mm^2^/s, *D* ^∗^ = 10·10^−3^ mm^2^/s, *f* = 0.05,
- Medium perfusion as *D* = 1.5 · 10^−3^ mm^2^/s, *D* ^∗^ = 15 · 10^−3^ mm^2^/s, *f* = 0.30,
- High perfusion as *D* = 1·10^−3^ mm^2^/s, *D* ^∗^ = 60·10^−3^ mm^2^/s, *f* = 0.30.

Noise was added to the simulated signals at signal‐to‐noise ratios (SNR) of 15, 30, 50, and 80 dB so that

(2) $$S\left(b\right)=\sqrt{S{\left(b\right)}^{2}+{\left[\frac{S\left(0\right)}{SNR}*\left(n_{R}+i\cdot n_{I}\right)\right]}^{2}}$$

where *n_R_* and *n_I_* are Gaussian distributions with unitary standard deviation (*n_R_,n_I_* ∼ N(0,1)), representing noise. After adding noise, the signal intensities simulated at each *b*‐value *S*(*b*) were normalized against the signal value with no diffusion gradient weighting (*S*(0)).

#### 2.1.2 Data fitting

A segmented fitting approach^26^ was adopted to derive IVIM parameters with a threshold equal to 200 s/mm^2^, commonly accepted in the literature.^2^, ^21^ Specifically, a linear fit for *D* was performed using the natural logarithm of the mono‐exponential diffusion component of the IVIM signal *S*(*b*)*/S*(0) = (1 − *f*)*e* ^−^ *^bD^*, limited to *b*‐values greater than the threshold. Then, *f* was estimated by extrapolating the previous linear fit back to *b* = 0 s/mm^2^ and employing *f* = (*S*(0)−*S*(*int*))*/S*(0), where *S*(int) is the y‐intercept of the fit and *S*(0) is the signal at *b* = 0 s/mm^2^. Negative estimates of perfusion fraction were discarded because unphysical and due, in general, to non‐Gaussian diffusion and noise effects.^2^ The estimated *D* and *f* were finally inserted into the biexponential decay equation (Equation 1) and *D* ^∗^ was estimated using a trust‐region, non‐linear least squares curve fitting method, exploiting only *b*‐values below the considered threshold. In order to yield only pathophysiological meaningful results, white matter and gray matter D^∗^ was constrained to [0, 0.1] mm^2^/s,^27^ while *D* ^∗^ values for the Gross Tumor Volume (GTV) were constrained to [0, 0.2]. The starting value for *D* ^∗^ was set to 0 mm^2^/s.

#### 2.1.3 Optimization strategy

To enable the optimization of the IVIM model, the errors pertaining IVIM parameters were combined in the sum of the relative errors, as done in previous studies.^19^, ^23^ The best *b*‐values distributions were chosen to be the ones that minimized the overall relative error *σ_tot_* = *σ_f_* + *σ_D_* + *σ_D_* ∗, with $\sigma_{x}=\sqrt{1/N\sum_{i=1}^{N}{\left(X_{i}-X\right)}^{2}}/X$, where *X* represents the true values of *D*, *f*, and *D* ^∗^, *X_i_* the fitted values of *D_i_*, *f_i_* , and *D_i_* ^∗^ at the *i*‐th iteration, and *N* the total number of iterations.

The candidate *b*‐values were constrained within the range [0, 1500] s/mm^2^ and the maximum number of *b*‐values was limited to 13. The upper *b*‐value limit was chosen to ensure that the signal decay due to gaussian diffusion weighting in the diffusion compartment was well approximated by mono‐exponential model, as reported in^21^, ^28^. The maximum number of *b*‐values was set to 13 to reach a *b*‐value distribution which results in a clinically feasible acquisition time and which represents an intermediate number of *b*‐values with respect to recent publications, as those cited in Chabert et al.^14^

The optimization procedure started with three fixed initial *b*‐values: 0, 200, 1500 s/mm^2^, where *b* = 0 s/mm^2^ corresponds to no diffusion weighting, 200 s/mm^2^ is the threshold of segmented fitting procedure and 1500 s/mm^2^ is the chosen upper limit.^28^ Since the fitting procedure consisted in a segmented approach, candidate *b*‐values to be added to the initial set were distinguished in below (i.e. *b*‐low, range [0, 190] s/mm^2^, step: 10 s/mm^2^) and above (i.e. *b*‐high, range [300, 1400] s/mm^2^, step 100 s/mm^2^) the threshold, thus resulting in 19 candidate *b*‐values *<*200 s/mm^2^ and 12 *>* 200 s/mm^2^. This choice reflected the strategy of sampling more *b*‐values in the range where the targeted IVIM effect is dominant (*<*200 s/mm^2^).

Starting from the initial set of *b*‐values, the overall relative error (*σ_tot_*) of each *b*‐values distribution obtained by adding one *b*‐value at the time was computed over 2000 simulations. At each iteration, the new optimal candidate distribution was defined by adding the *b*‐value that minimized *σ_tot_*. The chosen *b*‐value was then removed from the list of candidate *b* values for the next iteration to generate an array of unique *b*‐values, without repetitions. This process was repeated until the number of selected *b*‐values reached 13. The whole procedure was repeated 100 times to get 100 optimized *b*‐values distributions and to evaluate the frequency with which each *b*‐value occurred in a simulation. The underlying assumption of the optimization procedure is that the most frequent *b*‐values represent the best choice for IVIM parameters estimation. The final, optimal, distribution was given by the most frequent 13 *b*‐values identified over the 100 repetitions, considering as frequency the sum of the relative frequencies for all SNR levels. The optimal sets were characterized by 5 *b*‐values in the range [0, 190] s/mm^2^ and 5 *b*‐values in the range [300, 1400] s/mm^2^. The three predefined initial *b*‐values (0, 200, and 1500 s/mm^2^) were then added to these 10 *b*‐values.

#### 2.1.4 Experiments

Relative errors (*σ_tot_*, *σ_D_*, *σ_D_* ∗, *σ_f_*) of the optimized *b*‐values distributions were compared to a scheme with the same number of linearly distributed *b*‐values b‐lin(13b) = 0 30 60 90 120 150 200 400 600 800 1000 1200 1500 s/mm^2^. A backward elimination procedure was implemented to evaluate the feasibility of reducing the number of *b*‐values for a simplified IVIM model,^7^, ^29^, ^30^ and to compare this with a clinical procedure implemented at the National Center for Oncological Hadrontherapy (CNAO, Italy) in which 7 non‐optimized *b*‐values are currently acquired.^7^, ^11^, ^12^ In particular, the latter comparison is motivated by the possibility of deriving considerations that can be extended to all clinical settings in which, similarly to CNAO, reduced and non‐optimized DWI protocols are used, as reported by.^31^ The backward elimination of *b*‐values consisted in eliminating the least frequent b‐value in either the low ([0, 200] s/mm^2^) or high ([200, 1500] s/mm^2^) range. Starting from the obtained optimized distributions, at each iteration, the least frequent b‐value that minimized the overall relative error on simulations was removed. This procedure was repeated until the number of b‐values was equal to 4 – including the predefined initial *b*‐values of 0, 200, and 1500 s/mm^2^ and an additional one. Relative errors of a model with 7 optimal *b*‐values were then compared with a non‐optimal *b*‐values distribution as that currently used at CNAO, b‐CNAO(7b): [0 50 100 150 200 400 1000] s/mm^2^ and with a linearly distributed *b*‐values set b‐lin(7b): [0 60 120 200 600 1000 1500] s/mm^2^. In addition to b‐CNAO(7b) as reference set from the literature^7^, ^11^, ^12^, an optimal scheme of 14 b‐values (b‐lit(14b)) defined in the literature by Chabert et al.^14^ was also used for comparison.

Median values and interquartile ranges were computed for all IVIM parameters (*D*, *f*, and *D* ^∗^) in each perfusion regime (Low, Medium, High) for the different *b*‐values set, to evaluate IVIM parameters dependence on the number of *b*‐values.

For each perfusion regime, possible differences in the relative errors and in the IVIM parameter estimates acquired with different *b*‐value distributions were assessed with a Friedman test, applying a Bonferroni correction to account for multiple comparisons (*α* = 0.01).

Finally, the effect of optimized *b*‐values for Low, Medium, and High perfusion values was evaluated by performing a cross‐regimes analysis (e.g., by computing relative errors and IVIM parameters of the Low perfusion regime when using *b*‐values optimized for the High perfusion regime) for the optimal set of *b*‐values b‐opt(13b). This aimed at evaluating a unique set of *b*‐values in a clinical context when the tissue perfusion regime is unknown, as in case of our prospective acquisition protocol on patients with skull base chordoma tumor.

### 2.2 In vivo acquisitions

#### 2.2.1 DWI data acquisition

In vivo diffusion data were acquired on five healthy volunteers and five patients with skull‐base chordoma tumor prospectively selected before treatment with particle therapy at the National Center for Oncological Hadrontherapy (CNAO, Pavia, Italy) on a 3T MR scanner (MAGNETOM Skyra, Siemens Healthineers, Erlangen, Germany). The study was approved by the local Ethical Committee and informed consent was obtained. 7 *b*‐values of the b‐CNAO(7b) array (*b* = [0 50 100 150 200 400 1000] s/mm^2^) and 13 b‐values of the b‐opt(13b) array (*b* = [0 10 20 40 50 60 200 300 400 1200 1300 1400 1500] s/mm^2^) were used to acquire twice refocused spin echo DWI images (EPI, TE_7b_ = 58 ms, TE_13b_ = 65 ms, TR = 3300 ms, flip angle = 90^◦^, in‐plane resolution = 1.14 × 1.14 mm, slice thickness = 4 mm, matrix size = 200 × 200 × 19, slice gap = 0.8 mm) with full‐brain coverage. Diffusion‐weighted (trace) images were computed by playing the diffusion gradients along the three orthogonal directions of the scanner reference system. Acquisitions with b‐CNAO(7b) followed a NEX (number of averages) pattern of [2 2 2 2 3 3 5] empirically established per each corresponding b‐value, whereas a NEX = 2 was adopted for all b‐values of b‐opt(13b).

3D Slicer^32^ was used for a semi‐automatic masking of the brain based on intensity thresholding and further manual refinement. Segmentation of the brain into three main compartments (white matter, gray matter and cerebrospinal fluid) was obtained via 10 iterations of the MICO tool^33^ for MATLAB providing both the brain mask (refer to Figure S1) and the DW volume at *b* = 0 s/mm^2^ as inputs. On patients, the analysis was also conducted on the Gross Tumor Volume (GTV) defined before treatment with particle therapy.

#### 2.2.2 Retrospective analysis of *b*‐value sets

Data acquired with b‐CNAO(7b) were compared to data acquired with the set of 13 optimal *b*‐values found in the Medium perfusion regime to minimize cross‐regime variations (bopt(13b)‐Medium, see results on simulated data in Section 3.1) and with the array of 7 optimal *b*‐values obtained from backward elimination. IVIM parameter estimates were retrieved following the biexponential approach as explained in Section 2.1. Data from white and gray matter of each subject was used to estimate IVIM parameters and a Friedman test with a Bonferroni correction (*α* = 0.016) was performed for each parameter estimated with different b‐values sets for the volunteer and the patient populations. Also, a paired Wilcoxon rank‐sum test (*α* = 0.05) was used to compare parameter distributions between white and gray matter for each volunteer. On patients, the analysis of IVIM parameters was also conducted on the GTV.

## 3 RESULTS

### 3.1 Simulated data

**FIGURE 1:**
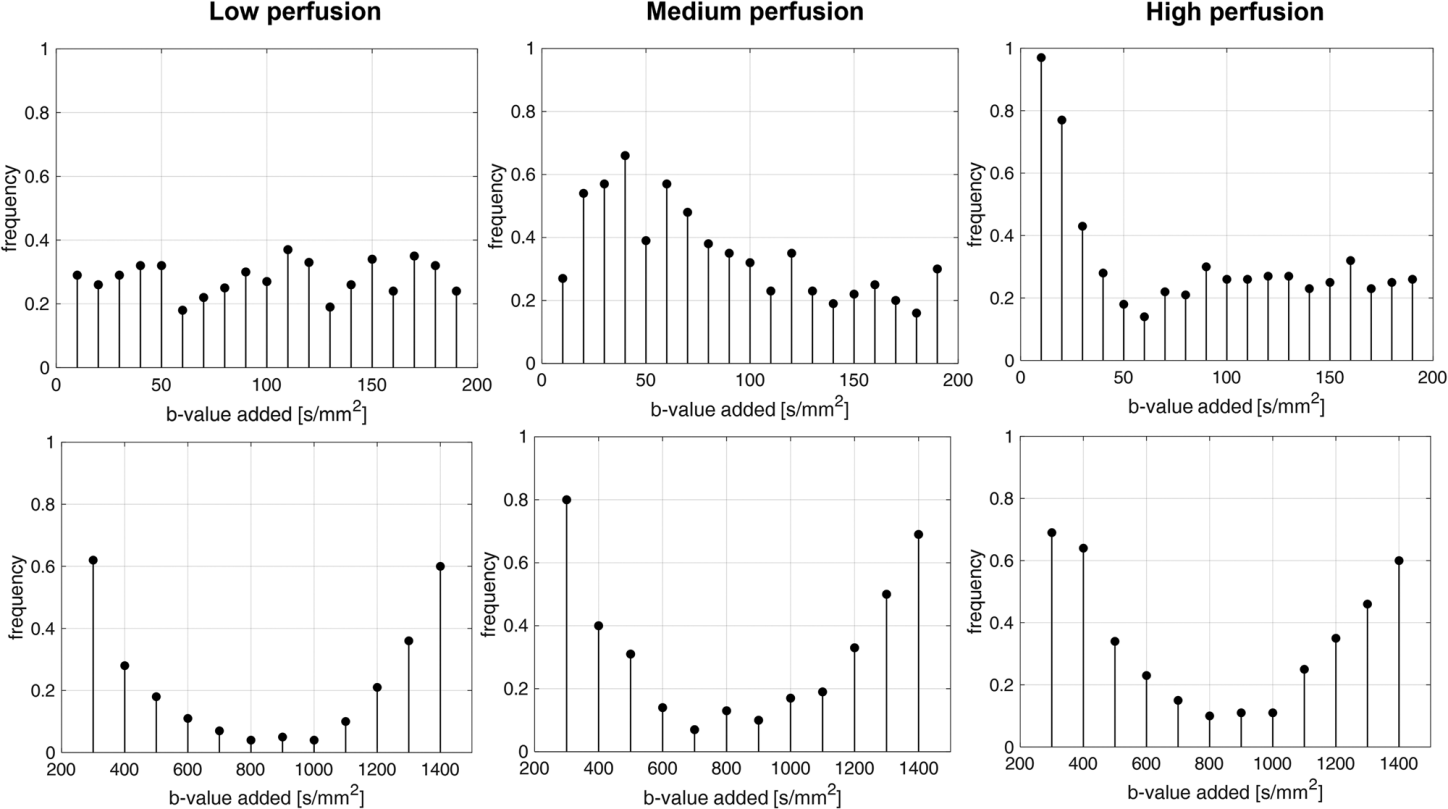
Frequency for candidate *b*‐values for SNR = 50 dB in low (*<*200 s/mm^2^, top row) and high (*>*200 s/mm^2^, bottom row) ranges for Low (left column), Medium (middle column), and High (right column) perfusion regimes.

Figure 1 represents the frequencies of candidate *b*‐values below (i.e. b‐low, range [0, 190] s/mm^2^, step: 10 s/mm^2^) and above (i.e. b‐high, range [300, 1400] s/mm^2^, step 100 s/mm^2^) the threshold, across all perfusion regimes. Figure 1 shows results obtained for SNR = 50 dB, which matches the SNR found in in‐vivo acquisitions (Section 3.2). Please refer to Figures S2–S4 for other SNR levels. For *b*‐values *<* 200 s/mm^2^, frequencies for *b*‐values *<* 50 s/mm^2^ resulted higher (*>*0.5) in the Medium and High perfusion regimes with respect to the Low perfusion regime. In the b‐high range (*>*200 s/mm^2^), the *b*‐values distributions showed the same trend among all perfusion regimes and SNR levels, with *b*‐values from 300 up to 500 s/mm^2^ and from 1200 up to 1400 s/mm^2^ showing higher frequencies. As such, according to these frequencies, the following optimal *b*‐values were selected for the three perfusion regimes:

- b‐opt(13b)‐Low = 0 10 30 50 60 150 200 300 400 1200 1300 1400 1500 s/mm^2^;
- b‐opt(13b)‐Medium = 0 10 20 40 50 60 200 300 400 1200 1300 1400 1500 s/mm^2^;
- b‐opt(13b)‐High = 0 10 20 30 40 50 200 300 400 500 1300 1400 1500 s/mm^2^.

**FIGURE 2:**
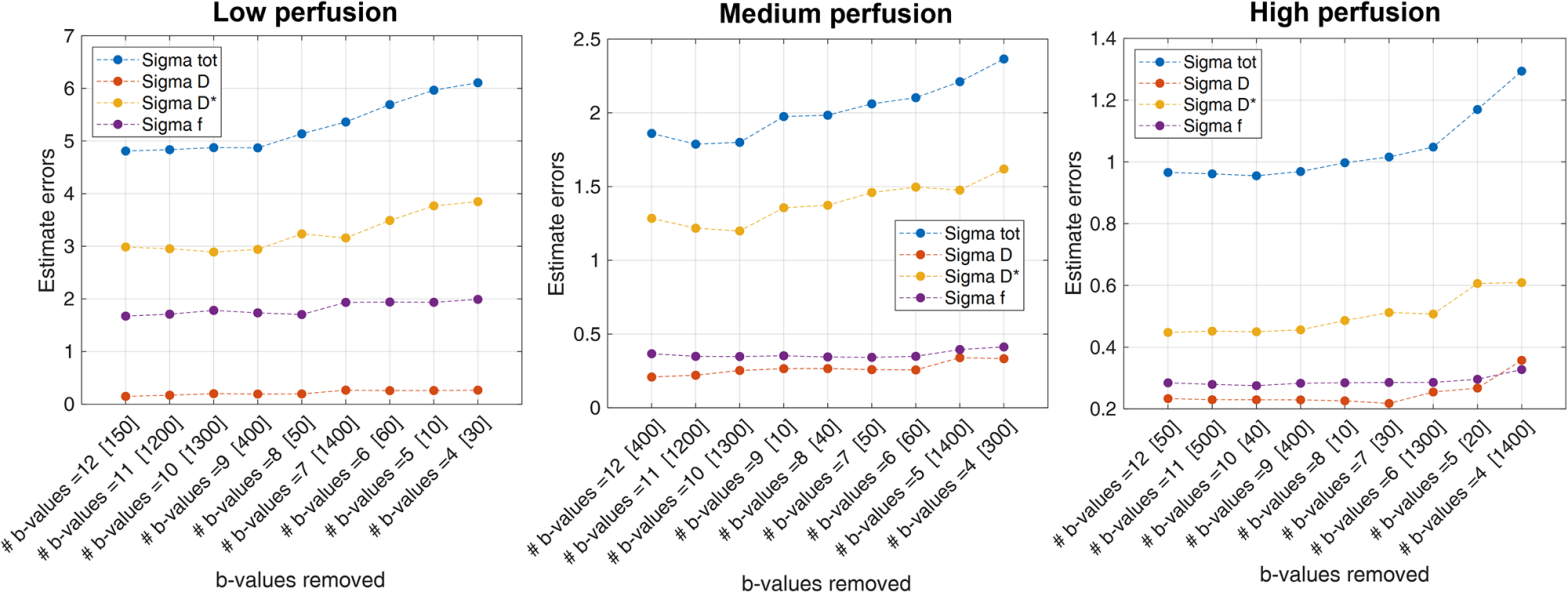
Errors versus backward elimination steps for Low (left column), Medium (middle column), and High (right column) perfusion regimes.

When eliminating non‐frequent b‐values during the backward elimination procedure (Figure 2), *σ_tot_* increased differently for the three perfusion regimes. Specifically, for Low and Medium perfusion regimes, the number of *b*‐values can be reduced to 9 and 10 while avoiding an error increment, whereas for the High perfusion regime, *b*‐values can be reduced up to a number of 6. *D* ^∗^ represented the IVIM parameter contributing the most to the error increment with respect to *D* and *f*. Over all the backward elimination steps, the mean contributes of *σ_D_*_∗_, *σ_f_*, and *σ_D_* to *σ_tot_* were 60%, 36%, and 4% in Low perfusion, 68%, 18%, and 14% in Medium perfusion, 47%, 28%, and 25% in High perfusion regime, respectively.

When considering 7 *b*‐values, the optimal sets of *b*‐values found through the backward elimination for each perfusion regime were:

- b‐opt(7b)‐Low = 0 10 30 60 200 300 1500 s/mm^2^,
- b‐opt(7b)‐Medium = 0 20 60 200 300 1400 1500 s/mm^2^,
- b‐opt(7b)‐High = 0 20 200 300 1300 1400 1500 s/mm^2^.

**FIGURE 3:**
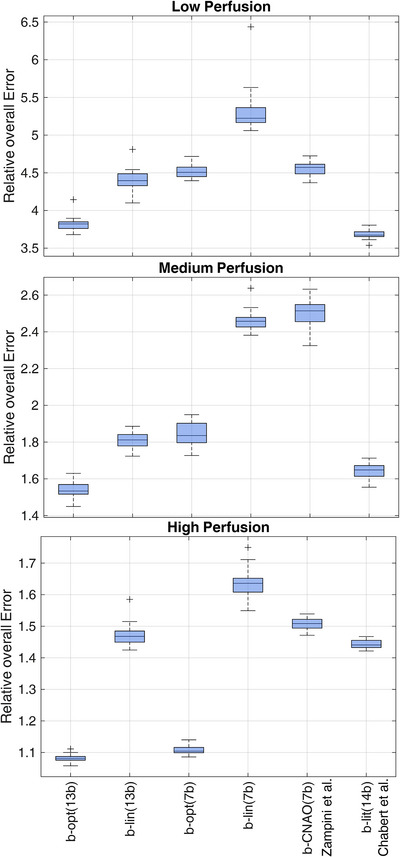
Relative overall error for different sets of *b*‐values in Low (top row), Medium (middle row), and High (bottom row) perfusion regimes.

The relative overall error *σ_tot_* for the optimal sets of *b*‐values (i.e., b‐opt(13b) and b‐opt(7b)), b‐lin(13b), b‐lin(7b), b‐CNAO(7b) and b‐lit(14b) is shown in Figure 3. In Low and Medium perfusion regime *σ_tot_* was not significantly different between b‐opt(13b) and b‐lit(14b), and resulted lower for these optimal sets with respect to the other b‐values sets. In High perfusion regime, *σ_tot_* in b‐opt(13b) was not significantly different from b‐opt(7b). In general, higher values of *σ_tot_* were observed for Low and Medium perfusion regimes with respect to the High perfusion regime.

**FIGURE 4:**
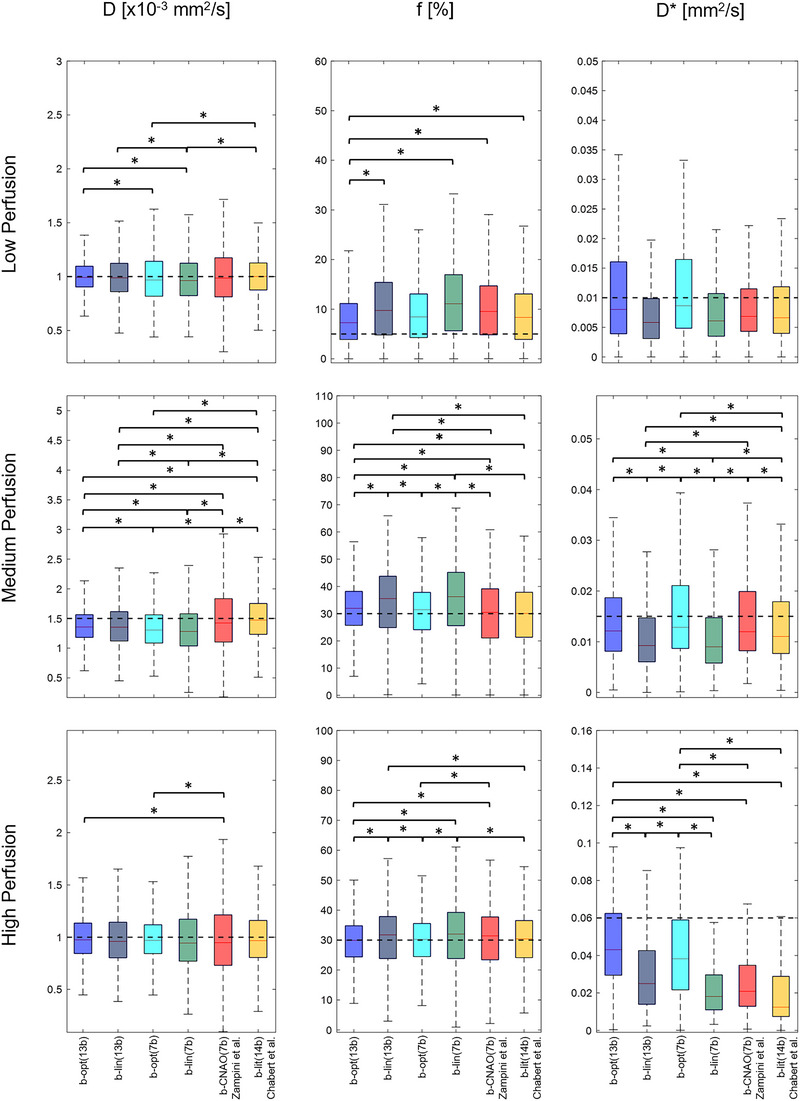
IVIM parameters estimation for the different sets of *b*‐values for Low (top row), Medium (middle row), and High (bottom row) perfusion regimes. The dashed horizontal line represents the true value adopted in the simulations. Significant differences (*α <* 0.01) are marked with *.

Regarding IVIM parameters (Figure 4 and Table S1, S2), D median and interquartile range values slightly varied when decreasing the number of *b*‐values, thus showing significant differences among optimized and non‐optimized *b*‐values sets. Similarly, *f* values were significantly different for most of the compared *b*‐values set. *D* ^∗^ median values increased towards the true value with b‐opt(13b), with significant differences among the *b*‐values sets for Medium and High perfusion regimes.

**FIGURE 5:**
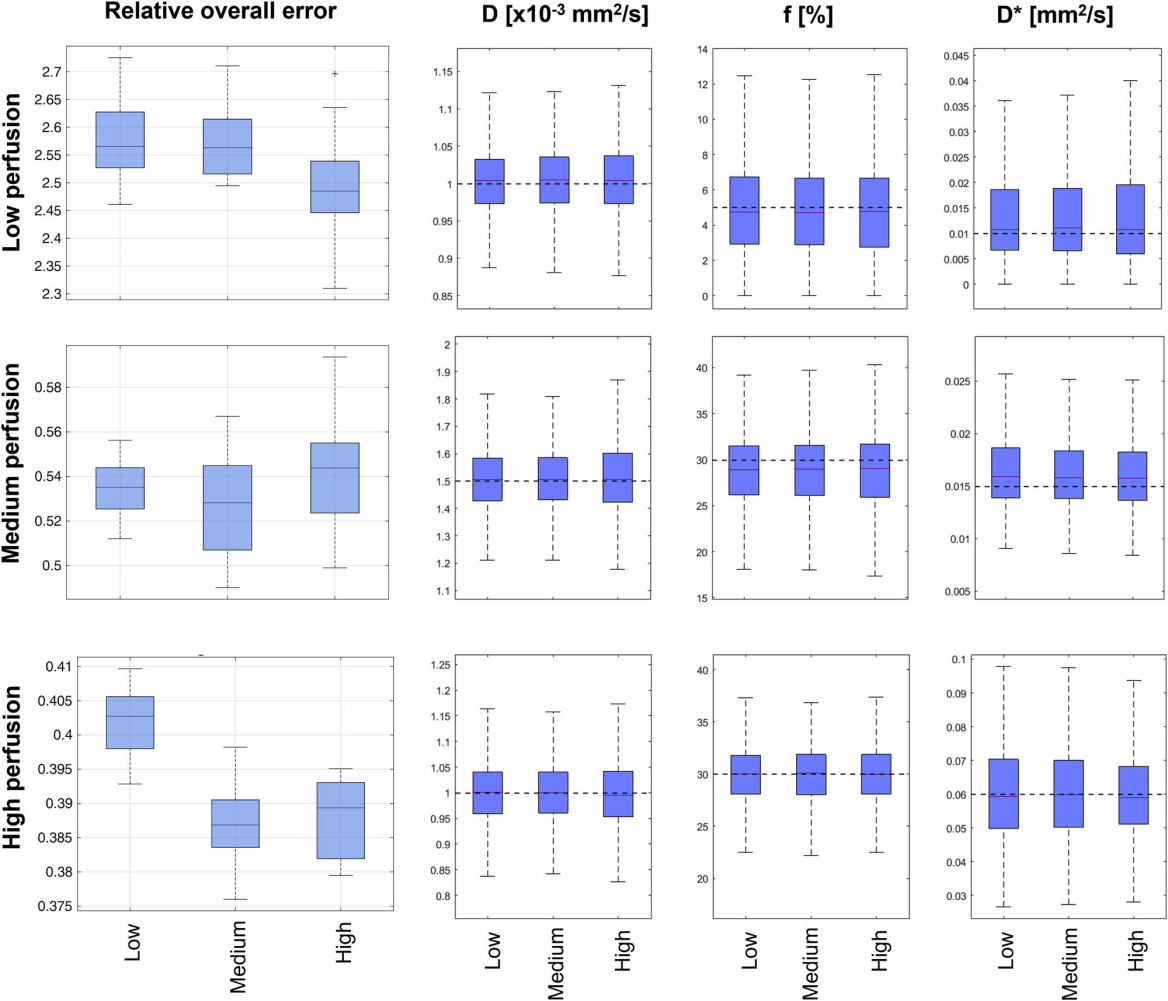
Cross‐regime performances. Relative overall error and IVIM parameters for b‐opt(13b) in each perfusion regime when using *b*‐values optimized for other perfusion regimes.

When evaluating cross‐regime performance (Figure 5), variations up to 5% were observed in the Low perfusion regime when using *b*‐values optimized for the High perfusion regime, as well as in the High perfusion regime when using *b*‐values optimized for the Low perfusion regime. Variations within 3% were instead present when using Low or High perfusion optimized b‐values in the Medium perfusion regime. Median values of IVIM parameters remained nevertheless comparable in the cross‐regime analysis with no significant differences (Figure 5 and Table S3).

### 3.2 In vivo acquisitions

**FIGURE 6:**
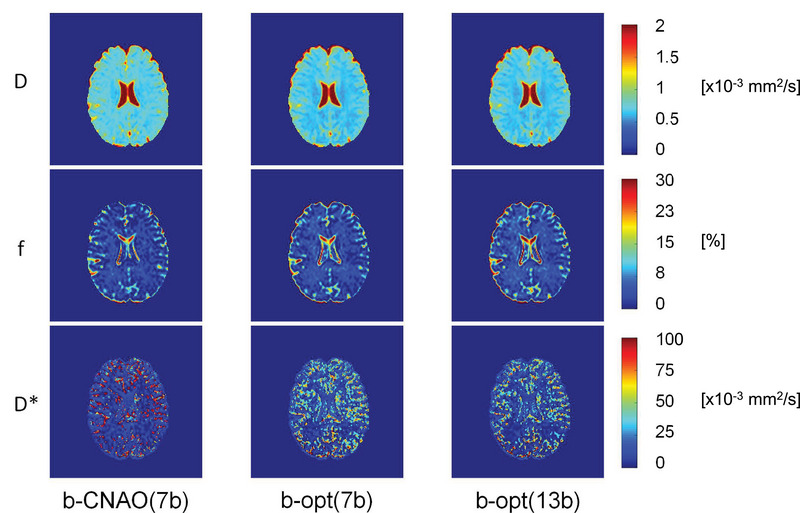
Example of the IVIM parameters maps computed with the three *b*‐values arrays for the first volunteer (see also Figure S6, for distribution of IVIM parameters for all the subjects).

Figure 6 reports an example of IVIM parameters maps computed using three different b‐value sets (b‐CNAO(7b), b‐opt(7b), and b‐opt(13b)) (Figure S5 reports a magnified section of ventricles, white, and gray matter). For in vivo acquisitions, b‐opt(7b) and b‐opt(13b) refer to *b*‐values set for Medium perfusion regime. The contrast between the tissues in the maps is visually increased by the use of the optimized b‐values arrays (b‐opt(7b) and b‐opt(13b)) with respect to the non‐optimal one (b‐CNAO(7b)), and Wilcoxon rank‐sum tests confirmed that white and gray matter distributions within the same b‐value set and for all the IVIM parameters have different median values (*p* *<* 0.001).

**FIGURE 7:**
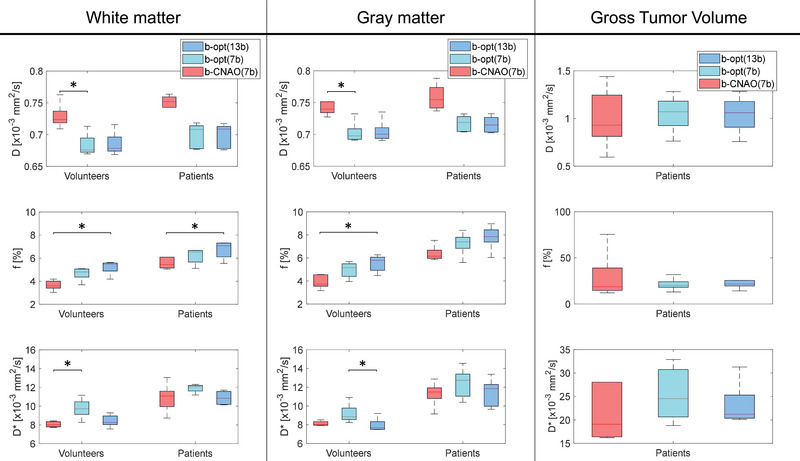
Boxplots reporting the distribution of IVIM parameters in white matter, gray matter, and Gross Tumor Volume for the different sets of *b*‐values employed (with NEX = 2). Significant differences among distributions (*α <* 0.016) are marked with *.

The distribution of IVIM estimates showed a dependency over the *b*‐values set used during acquisition: the selected array affected the estimation of all the IVIM parameters in white and gray matter of the volunteers, as reported in Figure 7. IVIM parameters (*D*, *f*, and D*) in white and gray matter (and GTV for patients) in the three sets of *b*‐values considered (b‐CNAO(7b), b‐opt(7b), and b‐opt(13b)) are reported in Table 1. Estimates for both tissues followed similar trends, with median *D*_b‐CNAO(7b)_ being higher than all the other median *D* values, as well with an increased median *f*_b‐opt(13b)_ with respect to *f*_b‐CNAO(7b)_, and with D*_b‐CNAO(7b)_ lower than D*_b‐opt(7b)_ in white matter and D*_b‐CNAO(13b)_ lower than D*_b‐opt(7b)_ in the gray matter. Values found in the GTV show a higher median value as well as a higher interquartile range with respect to parameter values in the white and gray matter, although the interquartile range is smaller for b‐opt(7b) and b‐opt(13b) than b‐CNAO(7b).

**TABLE 1:** IVIM parameters (*D* [×10^−3^ mm^2^/s], f [%], and D* [×10^−3^ mm^2^/s]) in white matter, gray matter, and Gross Tumor Volume in the three sets of *b*‐values considered (b‐CNAO(7b), b‐opt(7b), and b‐opt(13b)), reported as median value [interquartile range].

			*D* [×10^−3^ mm^2^/s]	*f* [%]	D* [×10^−3^ mm^2^/s]
		b‐CNAO(7b)	0.724 [0.018]	3.606 [0.610]	8.009 [0.555]
	White matter	b‐opt(7b)	0.676 [0.022]	4.807 [0.700]	9.706 [1.343]
Volunteers		b‐opt(13b)	0.679 [0.022]	5.491 [0.703]	8.251 [0.916]
		b‐CNAO(7b)	0.740 [0.017]	3.731 [0.996]	8.006 [0.392]
	Gray matter	b‐opt(7b)	0.698 [0.017]	5.1495 [1.099]	8.846 [1.229]
		b‐opt(13b)	0.701 [0.008]	5.797 [1.141]	7.674 [0.8289]
		b‐CNAO(7b)	0.752 [0.017]	5.439 [0.945]	11.086 [1.655]
	White matter	b‐opt(7b)	0.708 [0.036]	6.534 [1.005]	12.092 [0.957]
		b‐opt(13b)	0.709 [0.035]	7.075 [1.204]	10.849 [1.383]
		b‐CNAO(7b)	0.755 [0.032]	6.152 [0.715]	11.485 [1.125]
Patients	Gray matter	b‐opt(7b)	0.719 [0.024]	7.391 [0.956]	12.744 [2.360]
		b‐opt(13b)	0.715 [0.023]	7.841 [1.063]	11.837 [2.316]
		b‐CNAO(7b)	0.929 [0.433]	18.724 [24.426]	19.074 [11.664]
	Gross Tumor Volume	b‐opt(7b)	1.070 [0.256]	20.213 [6.066]	24.535 [10.109]
		b‐opt(13b)	1.060 [0.271]	21.537 [6.048]	21.192 [4.935]

## 4 DISCUSSIONS

In this study we investigated the search for an optimal *b*‐values scheme to derive accurate IVIM parameters in the brain with a segmented fitting procedure, by performing in‐silico simulations and in vivo acquisitions on healthy volunteers and patients.

An optimal set of *b*‐values was firstly investigated through in‐silico simulations of the IVIM signal at three different perfusion regimes, mimicking plausible patho‐physiological conditions in the brain. The optimization strategy aimed at minimizing the total relative error of the estimated IVIM parameters computed with a segmented fitting approach, similarly to Lemke et al.^23^ Such procedure was adopted to account for all IVIM parameters’ accuracy, without relying on costly in vivo acquisitions with empirically‐defined *b*‐values.^15^ Alternative optimization strategies such as that based on Cramér‐Rao Lower Bound theory^21^, ^34^ can be also considered in the future.

Our optimization method considered two ranges of *b*‐values defined with respect to the segmented fitting procedure threshold fixed at 200 s/mm^2^ (i.e. b‐low and b‐high ranges). The best *b*‐values candidates in the b‐high range did not depend on SNR and perfusion levels. This suggested that it is preferable not to choose uniformly distributed b‐values, but to group them near the lower and the upper limits. This result depends on the first step of the segmented fitting procedure, which consisted in the linear fit of the diffusion component of the IVIM signal and was confirmed on further tests performed on in vivo acquisitions (refer to Figure S7). Conversely, *b*‐values in the b‐low range varied depending on SNR and perfusion levels, with Medium and High perfusion regimes resulting in a denser sampling of *b*‐values where the perfusion effect is expected to be higher, that is in the 0 to 100 s/mm^2^ range. The different behavior in the Low perfusion regime can be explained by the fact that, due to low *f* (*f* = 0.05), the perfusion effect was minimal and the signal was approaching a mono‐exponential decay.

When reducing the number of *b*‐values with the backward elimination procedure, for Low and Medium Perfusion, where the contribution of D* had a starker impact on *σ_tot_*, *b*‐values in the b‐low range were removed in the last iterations of the procedure, suggesting that it is preferable to keep more low *b*‐values to compensate for fitting inaccuracies when the perfusion effect is minimal (i.e. mainly in Low perfusion regime). For High perfusion, where the contribution of D* on *σ_tot_* was lower, an equal number of *b*‐values in b‐low and b‐high was selected, due to the contribution of both perfusion and diffusion components. *σ_tot_* increased more sharply when going from 9 to 8 *b*‐values for Low perfusion, from 10 to 9 for Medium perfusion and from 6 to 5 for High perfusion. This indicates that the use of simplified IVIM protocols would require an a priori careful estimation of the perfusion properties of the targeted tissue, as errors strongly depend on perfusion regimes. In the literature, no agreement has been reached on the best amplitude and number of *b*‐values to be used in IVIM parameters estimation. Studies focusing on the liver, which can be considered a highly perfused tissue, showed that sets of 4 *b*‐values can be used if fast imaging is required.^19^ Studies in the brain instead presented a greater variability,^7^, ^15^ but it has been reported that at least 10 *b*‐values are commonly required for high quality IVIM experiments,^15^, ^23^ as also shown in our simulations.

The optimal *b*‐values sets were then compared with linearly distributed *b*‐values, with a clinical *b*‐values set currently performed at CNAO (b‐CNAO(7b)) and with the optimal *b*‐values set (b‐lit(14b)) defined by Chabert et al.^14^ The optimized schemes presented relative overall errors lower than those from linearly distributed sets and b‐CNAO(7b), suggesting that the clinical protocol would benefit from an optimization procedure. With respect to the literature optimal *b*‐values set proposed by Chabert et al.,^14^ no significant differences were observed in terms of relative error with respect to b‐opt(13b) in Low and Medium perfusion regime. Higher statistically significant error was instead observed for b‐lit(14b) in High perfusion regime, due to the fact that in Chabert et al. the optimal set was defined for low‐perfused tissues. The improved performance of optimized *b*‐values sets versus non‐optimized sets were also confirmed by significant differences observed in IVIM parameters, especially for Medium and High perfusion regimes, where the IVIM effect is more expected (Figure 4). With respect to IVIM parameters obtained with b‐lit(14b) in Medium perfusion, significant differences from the b‐opt(13b) parameters were found, with b‐lit(14b) appearing to perform better, even if lower overall errors were observed in b‐opt(13b). This could be explained by the greater amount of out‐of‐threshold values^27^ of b‐lit(14b) than b‐opt(13b); removing these values from the distributions led to median values of b‐lit(14b) closer to the true values adopted for simulations than b‐opt(13b). Specifically, out‐of‐threshold values were 0 (0%), 11 (0.55%), and 79 (3.9%) for b‐opt(13b) compared to 0 (0%), 96 (4.8%), and 474 (23.7%) for b‐lit(14b) in *D*, *f*, and D*, respectively. In light of this consideration, b‐opt(13b) is expected to be more robust than b‐lit(14b) in the estimation of meaningful IVIM parameters.

In order to define a unique set of b‐values for IVIM parameters mapping, to be used in our prospective acquisitions on patients treated at CNAO, the cross‐regime analysis highlighted the *b*‐values set optimized for Medium Perfusion as that with lower relative errors when used also in case of High or Low perfusion regime, although no significant differences were observed in IVIM parameters’ estimation (Figure 5 and Table S3).

Concerning in vivo acquisitions, the increase in the number of acquired *b*‐values from the original 7 used in clinical protocols at CNAO to the proposed set of 13 values in b‐opt(13b) directly translates into an increase of acquisition time (which also depended on different NEX values between the two acquisitions). Specifically, an increase of acquisition time by 56% was observed with respect to the current clinical procedure at CNAO (5 min 58 s vs. 3 min 2 s for b‐opt(13b) and b‐CNAO(7b), respectively), but anyway compatible with the clinical practice and with related literature studies^14^, ^15^, ^35^, ^36^.

We computed maps for IVIM parameters for five volunteers and five patients and found median estimates to be within a standard deviation from the averaged mean literature values reported in a recent publication.^14^ It should be noted that the mean values therein reported (white matter: *D*, *f*, and D* of 0.74 ± 0.12 ·10^−3^ mm^2^/s, 6.8 ± 4.9 %, and 25.3 ± 29.1 ·10^−3^ mm^2^/s; gray matter: *D*, *f*, and D* of 0.81 ± 0.15·10^−3^ mm^2^/s, 10.0 ± 7.4 %, and 21.9 ± 28.3 ·10^−3^ mm^2^/s) were computed from a collection of literature studies acquired with a variety of parameters, including the array of *b*‐values, number of averages, number of patients. For both white and gray matter in volunteers and patients, D_b‐CNAO(7b)_, f_b‐opt(13b)_, and D*_b‐opt(7b)_ were closer to the averaged literature values which, however, showed a strong variability and were computed from DWI images acquired with sets of different and not optimized b‐values. Within our analyses, in vivo values for white and gray matter were found to be closer to those of the Low perfusion regime, which corresponds to the anatomical features of the scanned tissues. Nevertheless, when targeting tissues the underlying physiology of which is not known (e.g. tumors), the optimal Medium perfusion *b*‐value set b‐opt(13b) seems to represent a valid choice. Since relative overall cross‐error for b‐opt(13b)‐Medium was comparable in the Low and Medium regimes, this b‐values set represents a good compromise for applications targeting both pathological and physiological tissues, such as tumor and normal tissue response to radiotherapy. This is confirmed by the IVIM values computed on the GTV of skull base chordoma patients: these were found to be higher than corresponding values for both white and gray matter in volunteers and patients, and values computed with the b‐opt(13b) and b‐opt(7b) *b*‐value sets show smaller variability with respect to values computed with b‐CNAO(7b). In particular, a noteworthy four‐fold reduction of the interquartile range is found for the perfusion fraction *f* computed with b‐opt(13b) and b‐opt(7b).

Also, simulations report that f values computed with b‐CNAO(7b) are higher than those defined for the Low Perfusion regime with respect to f values computed with b‐opt(7b) and b‐opt(13b), while in vivo data reported an opposite trend. As the simulations may not exactly match the in vivo underlying physiology, the trend of the parameters between simulations and experiments may not always correspond. However, precision with both b‐opt(7b) and b‐opt(13b) for *f* estimates was improved in both simulations and in vivo.

One limitation of the presented study is the lack of an optimized scheme of averages (NEX) that could modulate a NEX number to each *b*‐value. Nevertheless, we analyzed the signal variability by means of the Coefficient of Variation (CoV) over white and gray matter for each volunteer (refer to Figure S8). We observed that CoV decreased for higher *b*‐values, and that increasing the NEX number can contribute to lower CoVs, especially at low b‐values, as also suggested by Merisaari and colleagues^15^. As an increase in NEX corresponds to a further increase in acquisition time, we fixed NEX = 2 for our analysis, also considering that we observed limited variations when increasing NEX to 3 (Figures S8–S10). An additional limitation consists in the evaluation of a single fitting approach (i.e. segmented fitting) which can contribute to a different IVIM parameters’ estimation.^37^ From some preliminary analyses, the segmented fitting procedure provided more accurate estimates rather than a simultaneous fitting procedure (Figure S11). Further fitting methods should be anyway tested in the future along with extending this work to different field strengths and different sites, to gain further precision in IVIM studies. Moreover, as reported in^14^, part of the difficulty of working with IVIM acquisition is that there is no gold standard value to confirm which IVIM estimates values are to be expected. Our results improved precision when using an optimized b‐value distribution, which does not mean improved accuracy. Nevertheless, our work is put forward to provide an operator‐independent framework for the definition of an optimal b‐values set for IVIM parameter estimation in the brain by considering different perfusion regimes. Further comparison of IVIM perfusion‐related parameters, derived with optimized b‐values schemes, with pure perfusion sequences such as Dynamic Susceptibility Contrast (DSC) or Dynamic Contrast Enhancement (DCE) MRI, as suggested in Le Bihan^2^ and Zampini et al.^7^ will be considered, along with additional comparison/integration of the IVIM model with alternative multi‐compartment models, such as Diffusion Kurtosis Imaging (DKI).^34^, ^38^, ^39^

## 5 CONCLUSIONS

The effect of optimal b‐values schemes on DWI was investigated through in‐silico simulations and then tested on in vivo acquisitions on healthy volunteers and skull base chordoma tumor patients. The definition of an optimal *b*‐values set can improve the estimation of quantitative IVIM parameters computed with a segmented fitting procedure, with an increase of acquisition time still compatible with the clinical practice. This allows for setting up an optimized and reference approach that can be adopted for IVIM studies in the brain. We plan to extend the analysis on the prospective acquisitions that are currently ongoing at CNAO on patients with skull base chordoma tumor treated with charge particle therapy to evaluate changes in IVIM parameters before and after treatment.

## AUTHORS CONTRIBUTION

CP, MAZ, LM, GBu, and GBa contributed to conceptualization, formal analyses and paper writing; GF, LA, SI, GR, AI, EO contributed to data curation.

## CONFLICT OF INTEREST STATEMENT

All authors have no conflict of interests.

## Supporting information

Supplementary Information
