# Supplementary material for: Optimizing b‐values schemes for diffusion MRI of the brain with segmented Intravoxel Incoherent Motion (IVIM) model
Source: J Appl Clin Med Phys. 2023 Apr 9;24(6):e13986. doi: 10.1002/acm2.13986 (PMC10243330; doi:10.1002/acm2.13986)
Supplement: Supplementary file 1 — Supplementary Information [file ACM2-24-e13986-s001.pdf]

Optimizing b-values schemes for diffusion MRI of the brain with segmented Intravoxel Incoherent Motion (IVIM) model
# - Supplementary Material -

S1 In vivo data: example of tissue segmentation

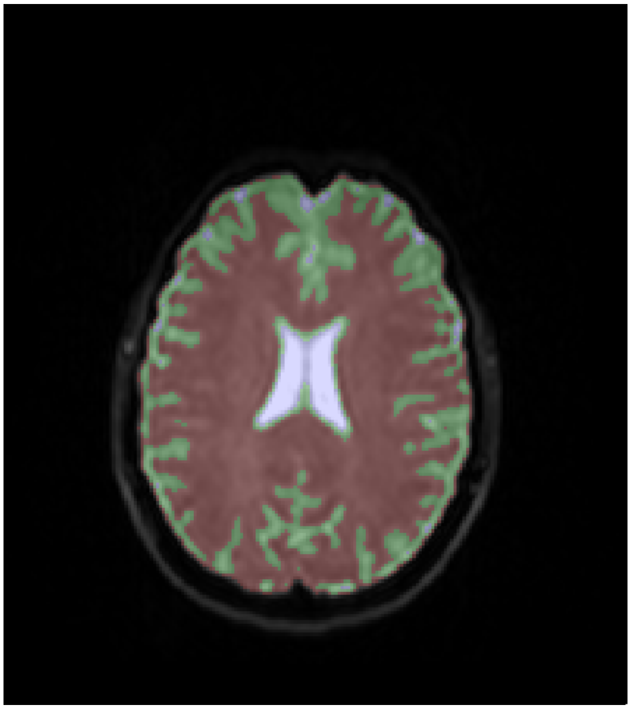

Figure S1: Brain tissues segmentation example into white matter, gray matter and cerebrospinal fluid shown as an overlay of a diffusion-weighted b=0 s/mm^2 image.

S2 In-silico data: b-values frequencies for different SNRs

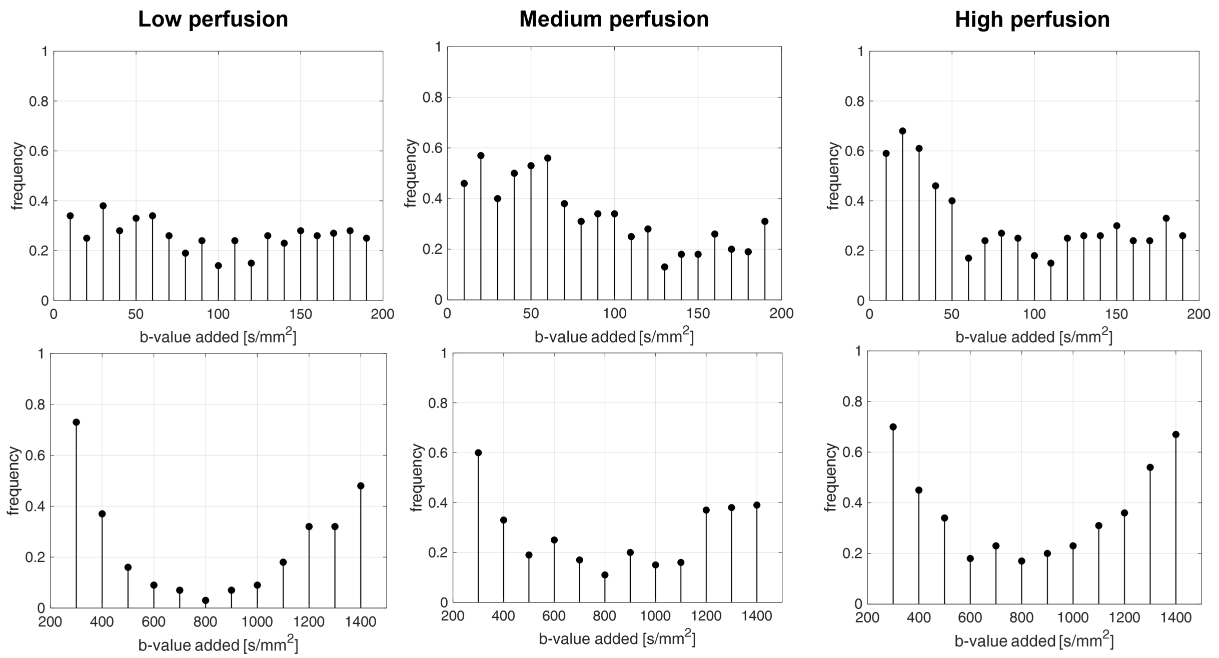

Figure S2: Frequency for candidate b-values for SNR=15dB in the low ($< 200s/mm^2$, top row) and high ($> 200s/mm^2$, bottom row) ranges for low (left column), medium (middle column) and high (right column) perfusion regimes.

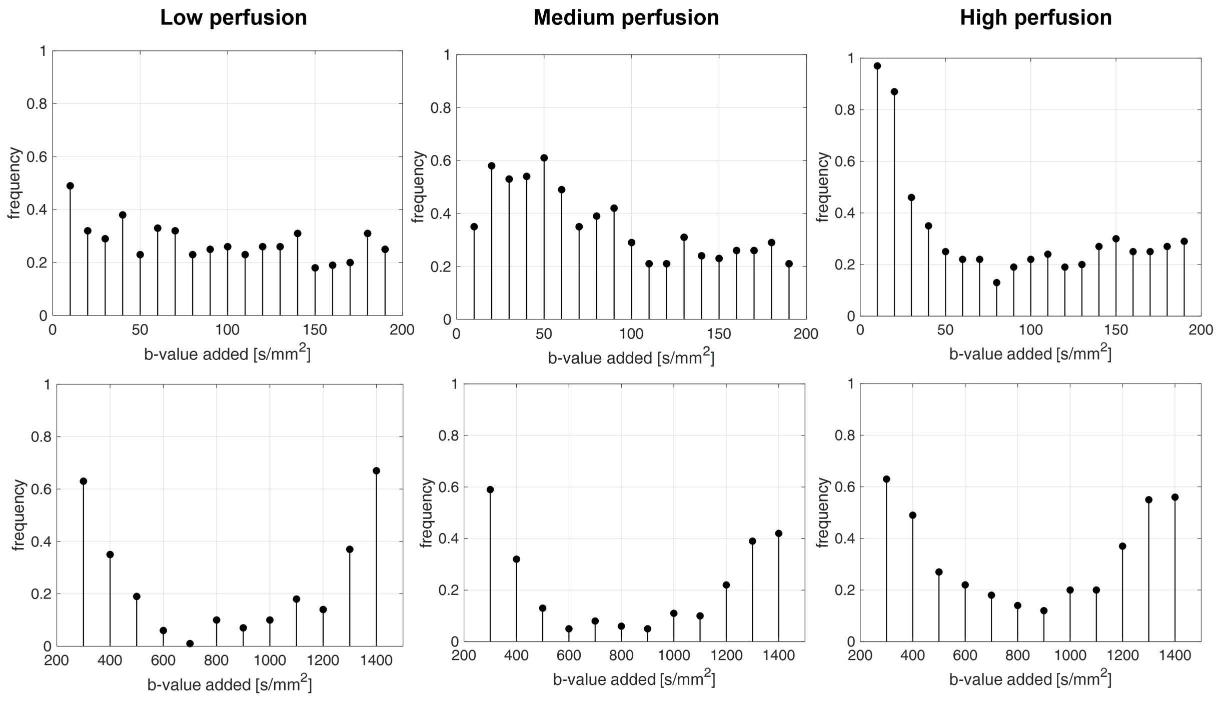

Figure S3: Frequency for candidate b-values for SNR=30dB in the low ($< 200s/mm^2$, top row) and high ($> 200s/mm^2$, bottom row) ranges for low (left column), medium (middle column) and high (right column) perfusion regimes.

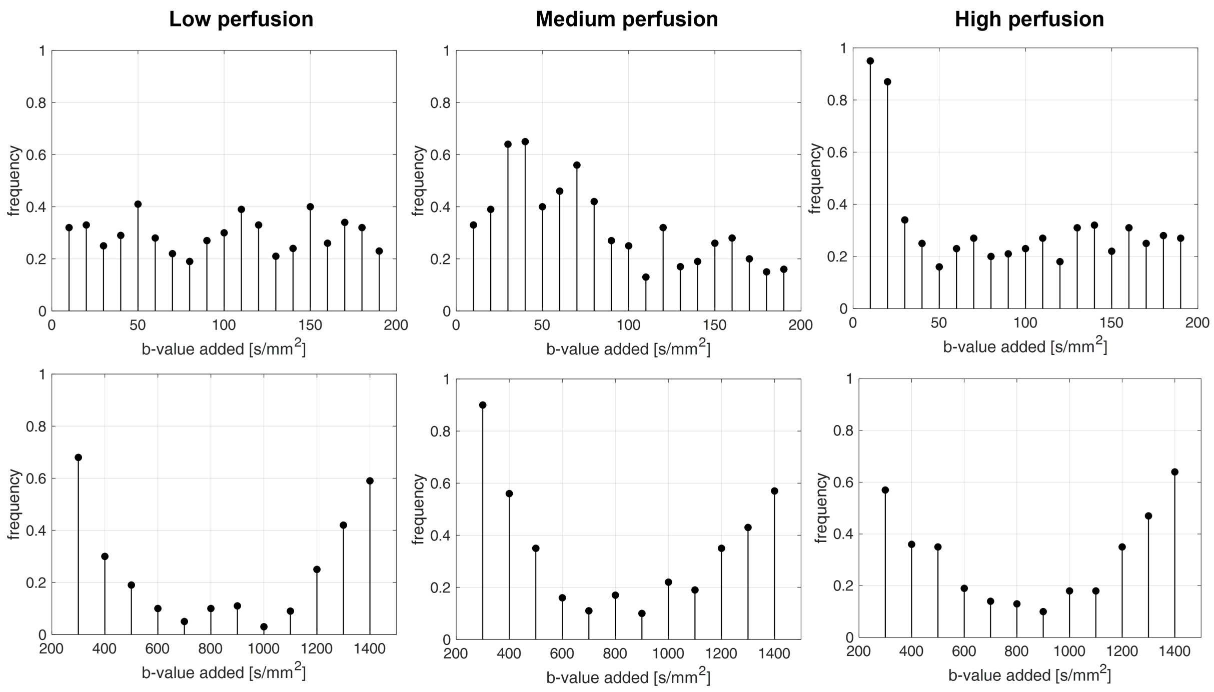

Figure S4: Frequency for candidate b-values for SNR=80dB in the low ($< 200s/mm^2$, top row) and high ($> 200s/mm^2$, bottom row) ranges for low (left column), medium (middle column) and high (right column) perfusion regimes.

S3 In-silico data: IVIM parameters estimation

Table S1: Median ± interquartile range of IVIM parameters for different b-values sets and different perfusion regimes (SNR=50dB).

	**Perfusion regime**	**D** $[x10^{-3}mm^2/s]$	**f** [%]	**D*** $[x10^{-3}mm^2/s]$
	$\text{low}_{TrueValue}$	**1**	**5**	**10**
	$\text{medium}_{TrueValue}$	**1.50**	**30**	**15**
	$\text{high}_{TrueValue}$	**1**	**30**	**60**
b-opt(13b)	low	0.99±0.19	8±8	8.75±12.21
	medium	1.41±0.42	31±10	12.11±10.50
	high	0.97±0.28	30±12	43.12±33.10
b-lin(13b)	low	0.98±0.26	10±11	5.81±6.83
	medium	1.41±0.51	35±19	9.33±8.71
	high	0.96±.33	31±15	25.12±28.61
b-opt(7b)	low	0.96±0.32	9±9	8.63±11.64
	medium	1.32±0.52	31±14	12.11±12.46
	high	0.96±0.27	30±12	38.22±27.36
b-lin(7b)	low	0.96±0.30	11±12	6.12±7.22
	medium	1.32±0.53	36±20	9.31±8.91
	high	0.94±0.40	32±16	18.12±18.71
b-CNAO(7b) Zampini et al.	low	0.98±0.36	10±10	6.91±7.23
	medium	1.40±0.78	30±18	12.03±11.70
	high	0.94±0.48	31±15	20.98±21.93
b-lit(14b) Chabert et al.	low	1.00±0.25	8±9	6.61±7.86
	medium	1.47±0.52	30±16	11.03±10.21
	high	0.96±0.35	30±12	12.42±21.34

Table S2: p-values for IVIM parameters. Significant differences are highlighted in bold.

	D	f	D*
Low Perfusion	**1.42e-05**	**2.04e-04**	0.1266
Medium Perfusion	**9.68e-58**	**2.64e-50**	**9.08e-35**
High Perfusion	**0.0037**	**1.02e-12**	**3.85e-18**

S4 In-silico data: Cross-regime analysis - IVIM parameters estimation

Table S3: p-values of the Wilcoxon rank-sum test for IVIM parameters computed in the cross-error analysis within in-silico simultations.

	D	f	D*
Low Perfusion	0.8918	0.7216	0.9061
Medium Perfusion	0.7831	0.9787	0.2896
High Perfusion	0.1442	0.4582	0.4756

S5 In vivo data: IVIM parameter maps

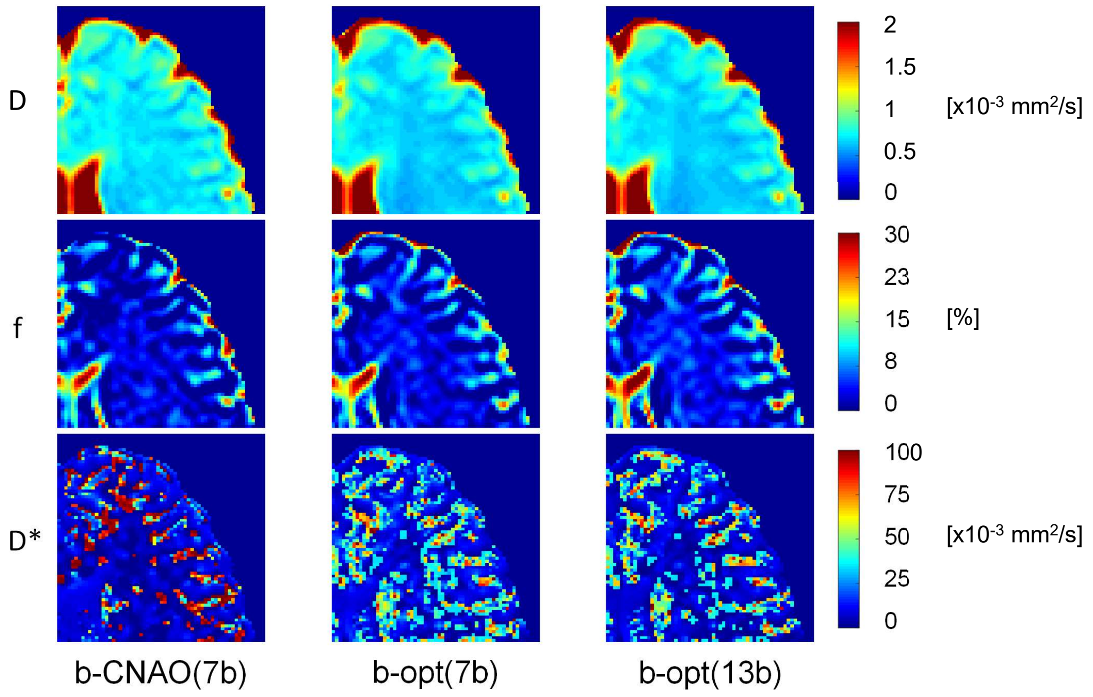

Figure S5: Magnified section for the IVIM parameters maps computed with the three b-values arrays.

S6 In vivo data: IVIM estimate comparison for each subject

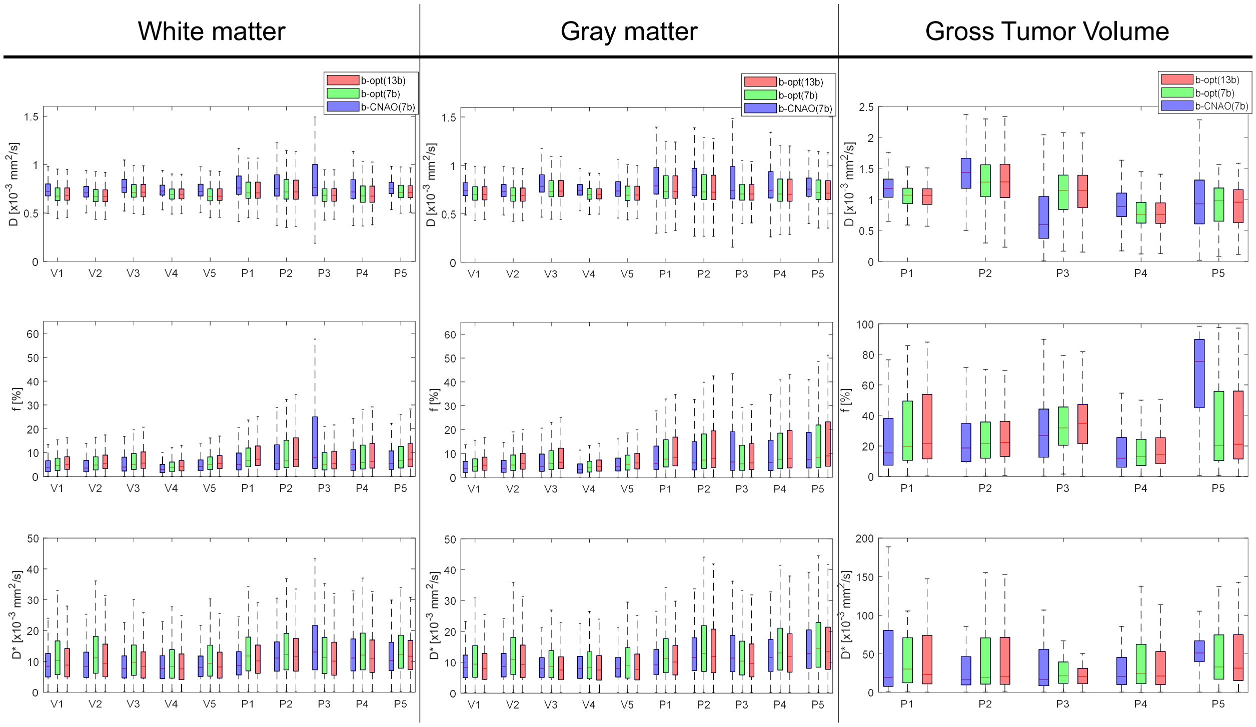

Figure S6: Boxplot reporting the distribution of IVIM parameters for volunteers (V) and patients (P) in white matter, gray matter, and Gross Tumor Volume for the different sets of b-values employed for each subject.

S7 In vivo data: b-values providing the lowest fitting error

To complement in-silico simulations, a further analysis was performed on in vivo acquisitions to assess the relative weight of each individual b-value on the estimation of D and f in the first step of the segmented approach. Two images acquired with b-values b = 800 and 1000 s/mm^2 were added to the images acquired with b-opt(13b) to check the impact of the additional b-values in parameter estimation over the threshold of 200 s/mm^2 and to avoid a gap in the b-value array between 400 and 1200 s/mm^2. Median intensity values for white and gray matter were used to compute D and f, and the residual sum of squares for every powerset of the b-value array used in the in vivo acquisitions. In order to find the most relevant b-value, for each subset of parameters with equal number of b-values (3 b-values, 4 b-values, 5 b-values, ...), we selected the one giving the smallest residual fit error and the occurrences in terms of an absolute cumulative frequency of each b-value were computed. Results are reported in Figure S7.

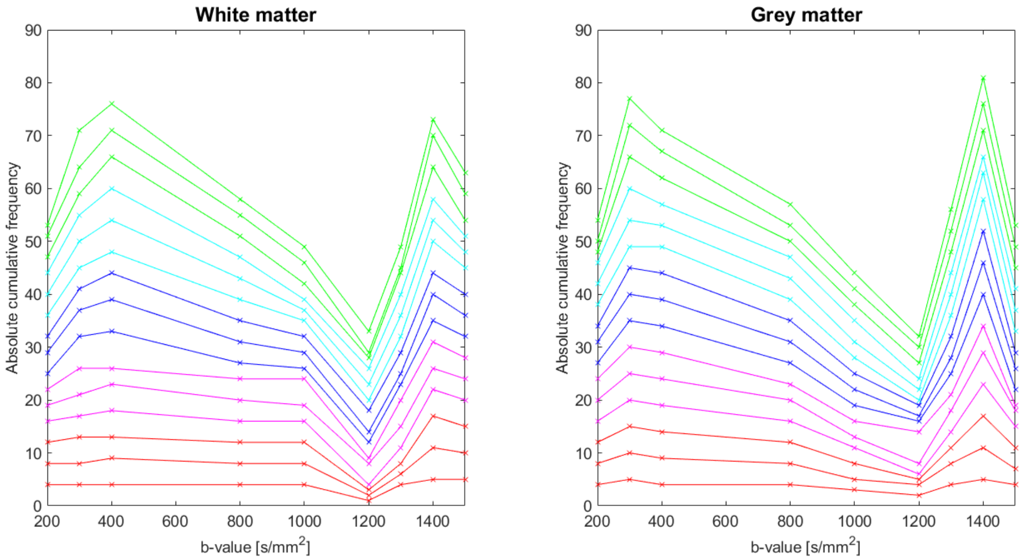

Figure S7: Occurrence of each specific b-value in an array made of b-opt(13b) and two additional b-values (b = 800 and 1000 s/mm^2) over the threshold of 200 s^2/mm minimizing the residual sum of square in the first step of the IVIM segmented approach for all the possible subsets of b-values. Results for different volunteer are reported with different colors for each volunteer and are added onto each other to show an absolute comulative frequency per each acquired b-value.

The absolute cumulative frequency for the retrospective selection of the b-values based on the smallest residual fit error confirms the selection of the b-values in the backward elimination process in the Medium perfusion regime within the simulations study. White and gray matter b-value frequency distributions shows two relative maximum in the b-values frequency for 300-400 and 1400 s/mm^2 (white matter) and for 300 and 1400 s/mm^2 (gray matter). These roughly reflect the trend of the backward elimination, which kept 1400 and 300 s/mm^2 as the last pivotal b-values for relative error minimization, while 400 s/mm^2 is eliminated early in the process. A further analogy between the two approaches can be seen with b-value = 1200 s/mm^2, which represents the absolute minimum in both the frequency distributions and is also eliminated early in the backward elimination process.

S8 In vivo data: signal variability over NEX

Three datasets for each volunteer were acquired repeating the DWI acquisitions for each b-value in b-opt(13b) and two additional b-values at b = 800 and 1000 s/mm^2. We computed the Coefficient of Variation (CoV) of $\log(S(b)/S(0))$ for white and gray matter (Figure S8) as a measure of signal variability to provide indications regarding the suggested number of averages (NEX).

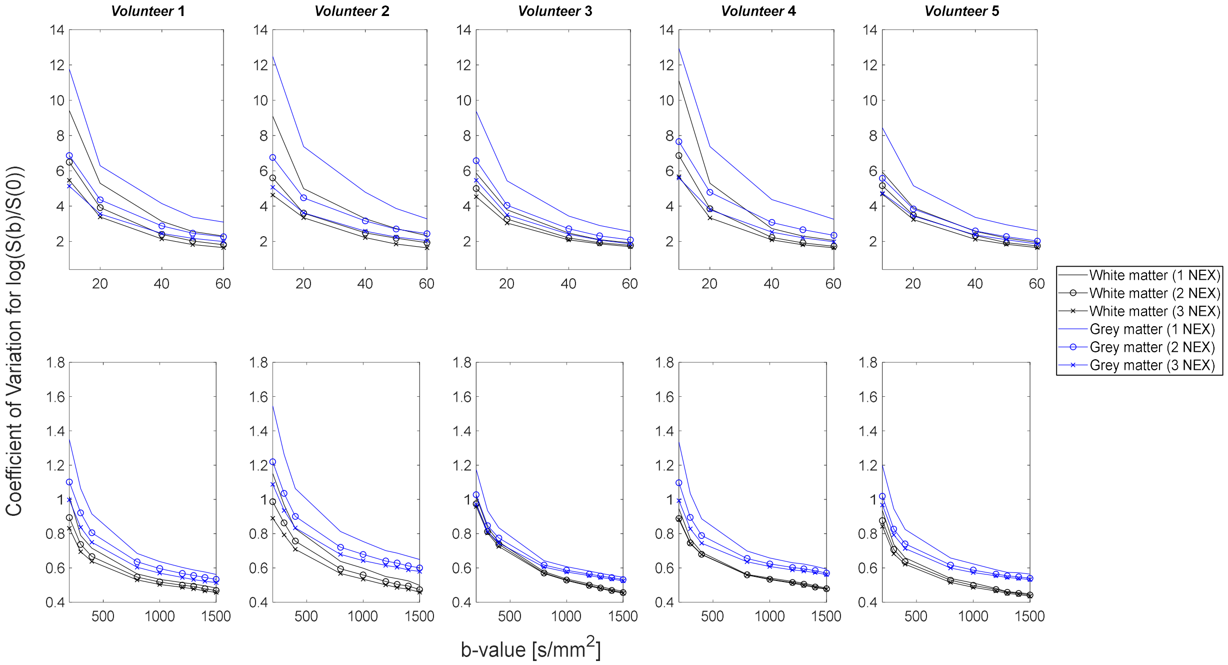

Figure S8: Coefficient of Variation of log(S(b)/S(0)) for white (black lines) and gray (blue lines) matter for each volunteer. The plot has been split in two parts (below and over the threshold of b = 200 s/mm^2) for visualization purposes.

The CoV decreases for high b-values and the acquisition of multiple NEXs corresponds to a decrease in the CoV, especially at low b-values. Nevertheless, the effect of NEXs on CoV becomes less relevant when higher b-values are used, making signal variability comparable between signals acquired with 1 or more averages. For a specific b-value, variability of the signal is higher for gray matter with respect to white matter. Considering that no relevant variations were observed when using 2 or 3 NEX, we decided to adopt NEX=2 as results to be presented in the manuscript. Nevertheless, here below, Figure S9 and S10 report the boxplot of the distribution of IVIM paramenters for NEX=1 and NEX=3, respectively.

Hereafter, boxplots reporting distributions of IVIM parameters among volunteers are reported for NEX=1 and NEX=3 (Figure S9 and Figure S10). In the manuscript, Figure 7 reports IVIM parameters for NEX=2. It should be noticed that statistical differences were computed on median values of IVIM parameters among volunteers.

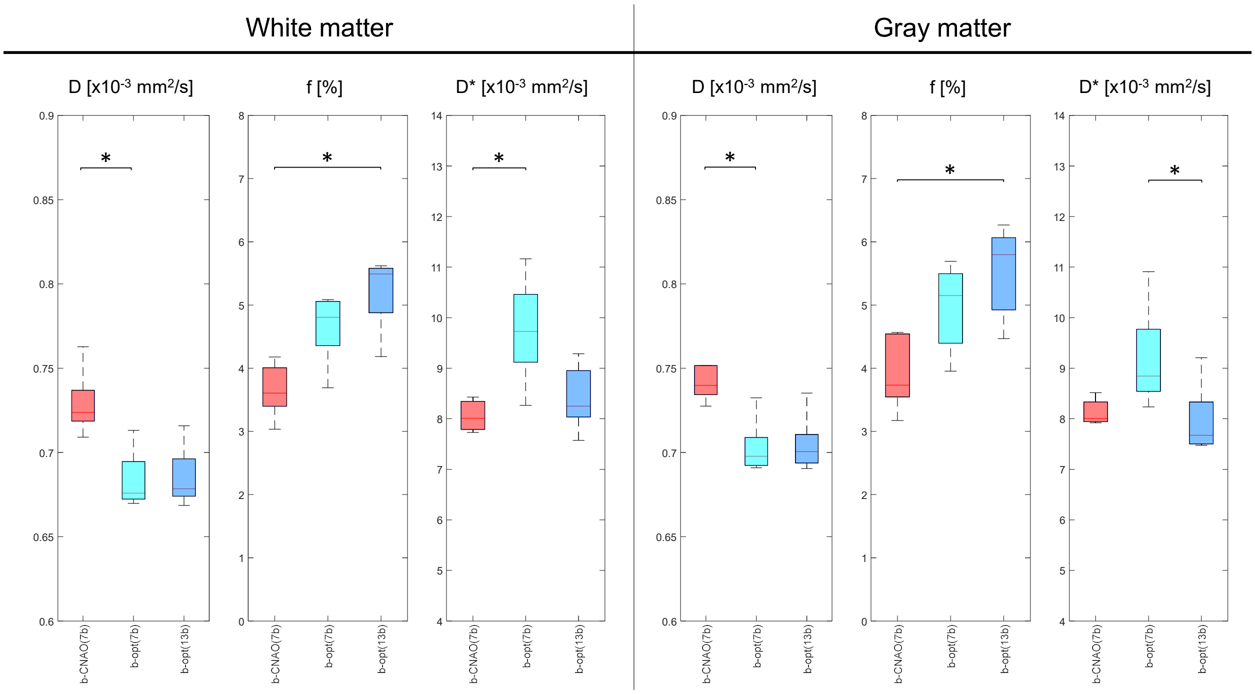

Figure S9: Boxplots reporting the distribution of IVIM parameters in white and gray matter for the different sets of b-values employed for NEX = 1. Significant differences among distributions ($\alpha < 0.016$) are marked with *.

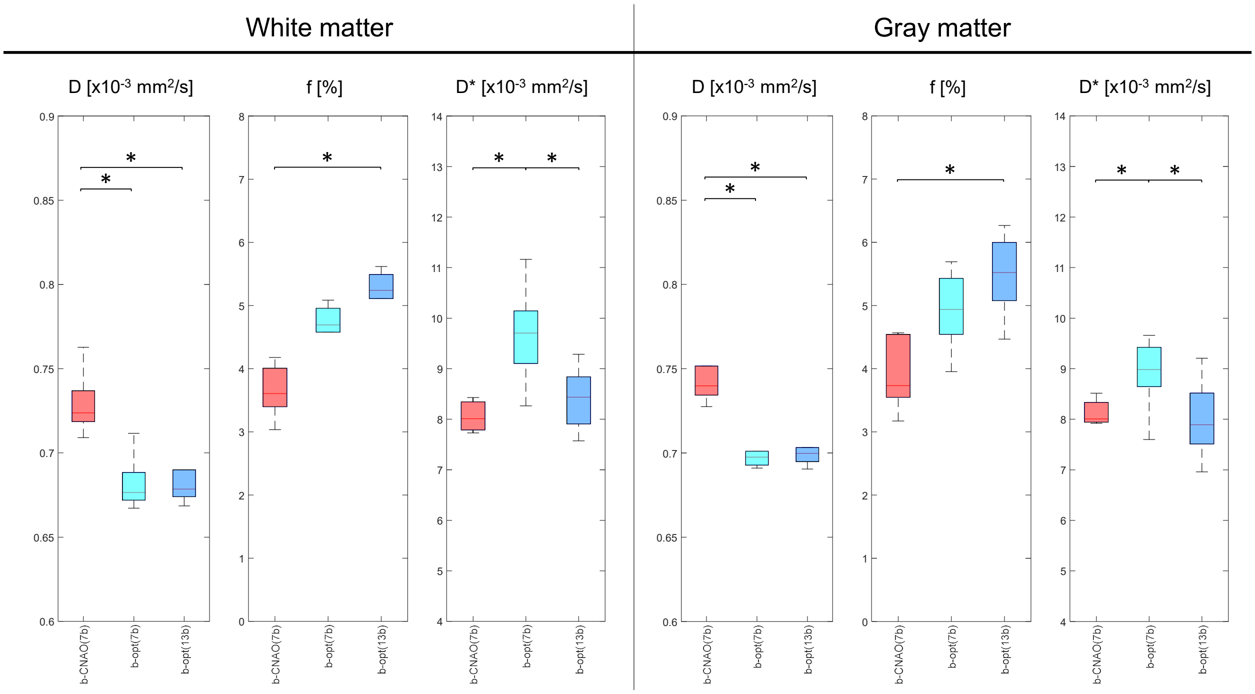

Figure S10: Boxplots reporting the distribution of IVIM parameters in white and gray matter for the different sets of b-values employed for NEX = 3. Significant differences among distributions ($\alpha < 0.016$) are marked with *.

S9 In-silico data: Segmented vs. Simultaneous fitting

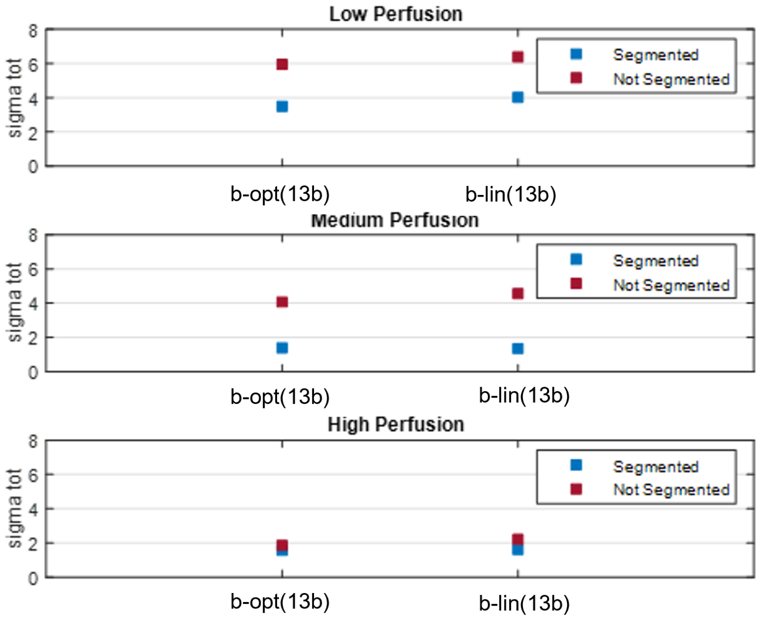

Figure S11: Mean values of σ_{tot} for 500 simulated signals for each perfusion regimes and optimal b-values schemes. The simultaneous fitting consisted in estimating all IVIM parameters together and using all the b-values without setting a threshold.
